# CRISPR–Cas technologies for precision genome editing in plants: advances, applications, and future perspectives

**DOI:** 10.3389/fpls.2026.1902159

**Published:** 2026-08-25

**Authors:** Syed Riaz Ahmed, Jahangir Khan, Iram Ijaz, Zeba Ali, Ijaz Rasul, Muhammad Aslam, Naeem Ud-Din, Yongming Liu, Muhammad Sayyam Tariq, Milan Skalicky, Daniel K. Y. Tan

**Affiliations:** 1 Pakistan Agricultural Research Council (PARC), Balochistan Agricultural Research and Development Centre (BARDC), Quetta, Pakistan; 2 Department of Botany, Faculty of Agriculture Sciences, University of Agriculture Faisalabad, Faisalabad, Pakistan; 3 Department of Plant Breeding and Genetics, Faculty of Agriculture Sciences, University of Agriculture Faisalabad, Faisalabad, Pakistan; 4 Department of Bioinformatics and Biotechnology, Government College University Faisalabad, Faisalabad, Pakistan; 5 National Nanfan Research Institute (Sanya), Chinese Academy of Agricultural Sciences, Sanya, China; 6 Division Plant Breeding and Genetics, Nuclear Institute for Agriculture and Biology (NIAB), Faisalabad, Pakistan; 7 Department of Botany and Plant Physiology, Faculty of Agrobiology, Food and Natural Resources, Czech University of Life Sciences, Prague, Czechia; 8 School of Life and Environmental Sciences, Plant Breeding Institute, Sydney Institute of Agriculture, Faculty of Science, The University of Sydney, Sydney, NSW, Australia

**Keywords:** base editing, CRISPR/Cas, functional genomics, genome editing, precision plant breeding, stress tolerance

## Abstract

Developing climate-smart crops with enhanced crop productivity, nutritional quality, resistance to biological and environmental stressors is vital for global food security. While hybrid breeding forms the cornerstone of modern crop improvement, conventional breeding approaches are limited by genetic barriers and prolonged breeding cycles. CRISPR–Cas based genome editing has revolutionized plant biology by allowing precise, efficient, and multiplex genetic modifications. This review provides a comprehensive synthesis of a recent advances in CRISPR–Cas technologies and their strategic applications in crop genetics and hybrid breeding. We summarize major genome-editing strategies, including gene knock-out, base editing (BE), knock-in, gene replacement, epigenome editing, and transcriptional regulation. Furthermore, we contrast stable, transient, and DNA-free delivery systems, highlighting ribonucleoprotein (RNP)-mediated delivery for minimizing off-target effects and avoiding transgene integration. We showcase how these technologies accelerate hybrid breeding by engineering male sterility systems, fixing heterosis, and generating high-throughput mutant libraries for trait discovery. Finally, we synthesize major bottlenecks in tissue culture-independent transformation and delivery systems, while outlining how emerging paradigms like *de novo* domestication and synthetic biology will shape the future of climate-resilient agriculture.

## 1 Introduction

Crop plants offer feed, food, energy, and many other raw materials essential for human and animal consumption, making agriculture a cornerstone of global society. It has been projected that by 2050, the universal population will reach about 9.6 to 9.7 billion, leading to an estimated 100–110% increase in crop demands compared with 2005 levels (Abdullah et al., 2025). Meeting this growing demand while maintaining nutritional quality is increasing challenging due to shrinking arable land, availability of limited water resources and the impacts of climate change. Thus, the introduction and application of advanced genomics and genome editing (GE) techniques have become critical for improving hybrid crop, enhancing productivity and supporting sustainable agriculture (Chen et al., 2019; Ahmed et al., 2024).

**Figure 1:**
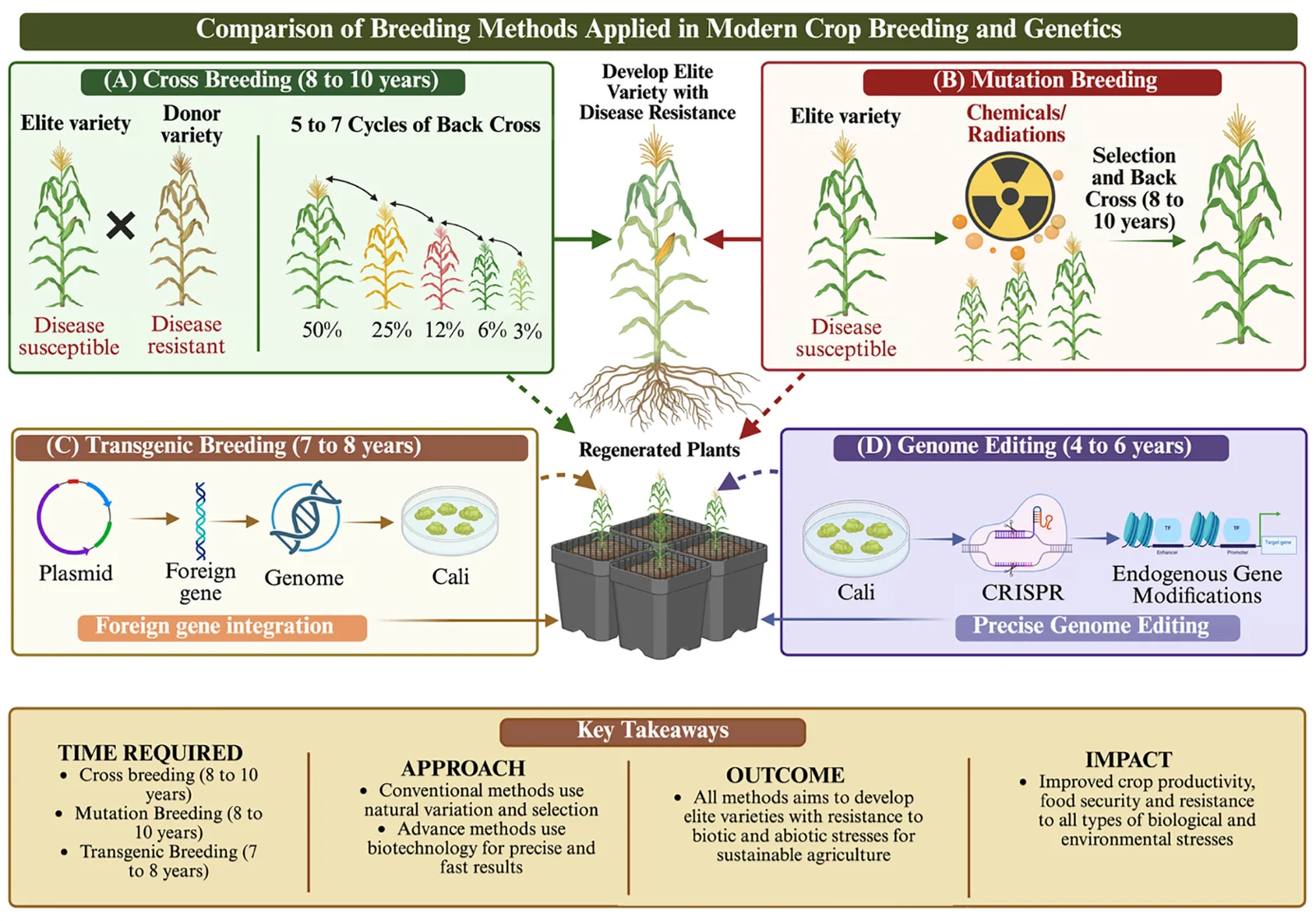
Comparative schematic of traditional and advanced crop breeding methodologies for elite cultivar development. A comprehensive overview highlighting timelines, underlying mechanisms, and technical complexity across four distinct breeding strategies aiming to engineer disease resistance or stress tolerance in agronomically superior crops. **(A)** *a*n elite, disease-susceptible line is crossed with a disease-resistant donor variety. This is followed by 5 to 7 cycles of phenotypic backcrossing to eliminate the genetic drag of the donor genome, progressively diluting the donor genome contribution (from 50% down to approximately 3%) while retaining the target resistance trait. This conventional workflow typically requires 8 to 10 years to stabilize an elite variety. **(B)** seeds or tissues of an elite, disease-susceptible line are exposed to physical (e.g., gamma radiations) or chemical (e.g., ethyl methanesulfonate) mutagens to induce random genomic modifications. Subsequent phenotypic selection and multi-generation backcrossing are required to isolate favorable mutants while purging deleterious mutations, requiring a total of 8 to 10 years. **(C)** a recombinant expression vector/plasmid harboring an exogenous target sequence (foreign gene) is introduced into the plant genome via Agrobacterium-mediated transformation or biolistics. The transformed cells are regenerated via somatic embryogenesis or tissue culture (Cali) to generate stable, transgenic plants carrying foreign gene integrations. This biotechnological approach spans 7 to 8 years. **(D)** explant tissues or undifferentiated embryogenic cells (Cali) are modified using CRISPR-Cas site-specific endonucleases. The CRISPR system induces precise double-strand breaks at pre-determined loci to introduce endogenous gene modifications (such as targeted knockouts via non-homologous end joining or precision edits). Because it avoids genetic drag and foreign gene integration, the workflow achieves precise modification within a compressed timeframe of 4 to 6 years.

Conventional crop improvement strategies, including hybrid breeding, hybridization, mutation induced breeding, and transgenic approaches, have contributed significantly to agricultural progress (Ud-Din et al., 2025; Kalogeropoulos et al., 2026). Hybrid breeding through genetic recombination increases genetic diversity and trait performance; however, the process is time-consuming and often requires 7–10 years to develop elite hybrids (Revathi et al., 2025; Sarun, 2026). Mutation breeding introduces genetic variation through physical or chemical mutagens, but this arbitrary nature of induced mutations necessitates extensive screening and makes trait selection laborious and inefficient (Pacher and Puchta, 2017; Ud-Din et al., 2025). Even with marker-assisted breeding, these approaches often fail to meet the accelerating demands of modern hybrid crop improvement (Revathi et al., 2025; Kalogeropoulos et al., 2026). Transgenic breeding enables the introduction of desirable genes across species barriers, but its widespread adoption remains constrained by regulatory restrictions and public concerns surrounding genetically modified organisms (GMOs) (**Figure 1**) (Behera et al., 2026).

The development of targeted double-strand breaks (DSBs) in DNA can increase gene-editing efficiency marked a turning point in plant biotechnology (Puchta et al., 1993). This finding led to the introduction of site-specific GE tools, like zinc finger nucleases (ZFNs), transcription activator-like effector nucleases (TALENs), and clustered regularly interspaced short palindromic repeats associated with Cas proteins (CRISPR/Cas) (Dong and Ronald, 2021). Although TALENs as well as ZFNs have already been applied successfully in crops, their complicated design, high cost, limited targeting flexibility, and variable off-target effects have restricted their widespread use in breeding programs (Younas et al., 2025; Li et al., 2026).

CRISPR/Cas systems, originated from bacteria’s adaptive immune mechanisms, have appeared as a transformative GE technology due to their efficiency, simplicity and cost-effectiveness (Doudna and Charpentier, 2014). In this system, a guide RNA directs the Cas endonuclease protein to a complementary genomic sequence, where precise DNA cleavage enables targeted gene modifications. The CRISPR/Cas9 was initially introduced for plant GE in 2013, successfully demonstrated in *Arabidopsis thaliana*, *Nicotiana tabacum*, rice, and wheat (Shan et al., 2013; Nekrasov et al., 2013; Li et al., 2013). Subsequent improvements, including alternative nucleases like Cpf1 (Cas12a) and base-editing (BE) approaches, have further enhanced editing precision and expanded the range of genetic modifications achievable in crops (Li et al., 2024). CRISPR/Cas application has allowed precise modifications of genes that are linked with important agronomic traits, including production, nutritional quality, and resistance to biological and environmental stresses. In the context of hybrid breeding, CRISPR/Cas facilitates rapid trait integration, elimination of undesirable alleles, and efficient development of elite parental lines. These advances significantly accelerate breeding cycles and improve the genetic potential of hybrid crops, offering a powerful solution to meet worldwide food demands and promote sustainable agricultural systems (Behera et al., 2026).

While numerous review articles have broadly detailed CRISPR-Cas applications in plants, they often treat general trait improvement and hybrid breeding as isolated topics (Chen et al., 2019; Ali et al., 2023; Basu et al., 2023). A critical knowledge gap remains in understanding how diverse genome editing modalities can be systematically integrated to revolutionize hybrid breeding pipelines. This review aims to bridge this gap by providing an extensive and integrative synthesis of CRISPR–Cas based GE technologies especially through the lens of hybrid plant breeding. Specifically, it critically examines diverse CRISPR-mediated strategies like gene knock-out, knock-in, gene replacement, BE, epigenome editing, transcriptional regulation using dCas9, and DNA-free editing approaches, evaluating their direct roles in accelerating the introduction of high-yielding, stress-resilient, and quality-improved hybrid crops (Sgro and Blancafort, 2020). Furthermore, recent breakthroughs in DSB-free genome editing, multiplex editing, epigenome engineering, precise DNA insertion (knock-in), AI-assisted guide RNA design, and advanced delivery systems have substantially expanded the CRISPR toolbox, yet an integrated assessment of their implications for hybrid crop development remains lacking (Kim et al., 2025). Addressing this gap, the present review provides a comprehensive and up-to-date synthesis of CRISPR–Cas technologies from the perspective of precision plant breeding, with particular emphasis on their application in accelerating hybrid breeding strategies. Moreover, this review highlights advances in editing reagent delivery systems, compares stable, transient, and RNP-based GE methods, and summarizes successful applications across major cereal and horticultural crops. Additionally, uniquely focuses on the use of CRISPR-Cas technologies for hybrid breeding acceleration, including male sterility systems, heterosis fixation, trait pyramiding, and rapid *de novo* domestication of wild relatives. By integrating recent progress, technical challenges, and future prospects, this review provides a novel framework for effectively incorporating CRISPR-Cas GE into modern plant hybrid breeding programs to advance sustainable crop improvement.

## 2 Modifications in plant genome via CRISPR/Cas

The CRISPR/Cas system is an adaptive immune system originating from archaea and bacteria that has been repurposed into versatile genome editing platform for plants. The system consists of CRISPR-derived RNA- guided nuclease that recognizes target DNA sequences adjacent to PAM and introduces site-specific DNA cleavage (Wei et al., 2026). Based on their effector complexes, CRISPR/Cas systems are classified into two major classes and six types, with Class 2 systems, including Type II (Cas9), Type V (Cas12), and Type VI (Cas13), being the most widely employed for plant genome engineering because they utilize a single multidomain effector protein (Makarova et al., 2025).

Among these, SpCas9 remains extensively used nuclease owing to its high editing efficiency and well characterized NGG PAM requirement (Nadeem et al., 2024). Continuous protein engineering has expanded the CRISPR toolbox through the development of Cas9 orthologues and engineered variants with altered PAM recognition, including SaCas9, NmCas9, StCas9, SpCas9-NG, xCas9, VQR-Cas9 and VRER-Cas9, substantially increasing the number of editable genomic loci in crop species (Alariqi et al., 2025; Swain et al., 2025; Shu et al., 2025). Likewise, Cas12 nucleases have broadened genome-editing applications by recognizing T-rich PAMs while simultaneously facilitating multiplex editing through their intrinsic crRNA-processing capability (Pandey et al., 2025; Han et al., 2024). Following CRISPR-mediated cleavage, genome modifications are generated through endogenous DNA repair pathways (Muzaffar et al., 2026; Moroi et al., 2025; Jin et al., 2025). Non-homologous end joining (NHEJ) is the predominant repair mechanism in plants and frequently introduces small insertions or deletions that disrupt gene function, making it the preferred strategy for targeted gene knockouts (Cui et al., 2019; Mansi and Danai, 2026; Vollen et al., 2025). In contrast, homology-directed repair (HDR) uses homologous DNA templates to mediate precise sequence replacement or targeted gene insertion (Vollen et al., 2025; Liang et al., 2025). However, HDR remains considerably less efficient in plants because of competition from NHEJ, limited donor-template availability and cell-cycle constraints (Jash et al., 2026; Gilbertson et al., 2025). Recent approaches including geminivirus-derived replicons, optimized donor-template delivery systems and chimeric guide RNA designs have improved HDR efficiency, although precise template-directed editing is still less routine than NHEJ-mediated mutagenesis (Luqman et al., 2025; Naveed et al., 2025). Collectively, advances in nuclease engineering together with improved manipulation of DNA repair pathways have considerably expanded the scope of CRISPR-mediated genome engineering in plants while providing the molecular foundation for the next generation of precision editing technologies.

**Figure 2:**
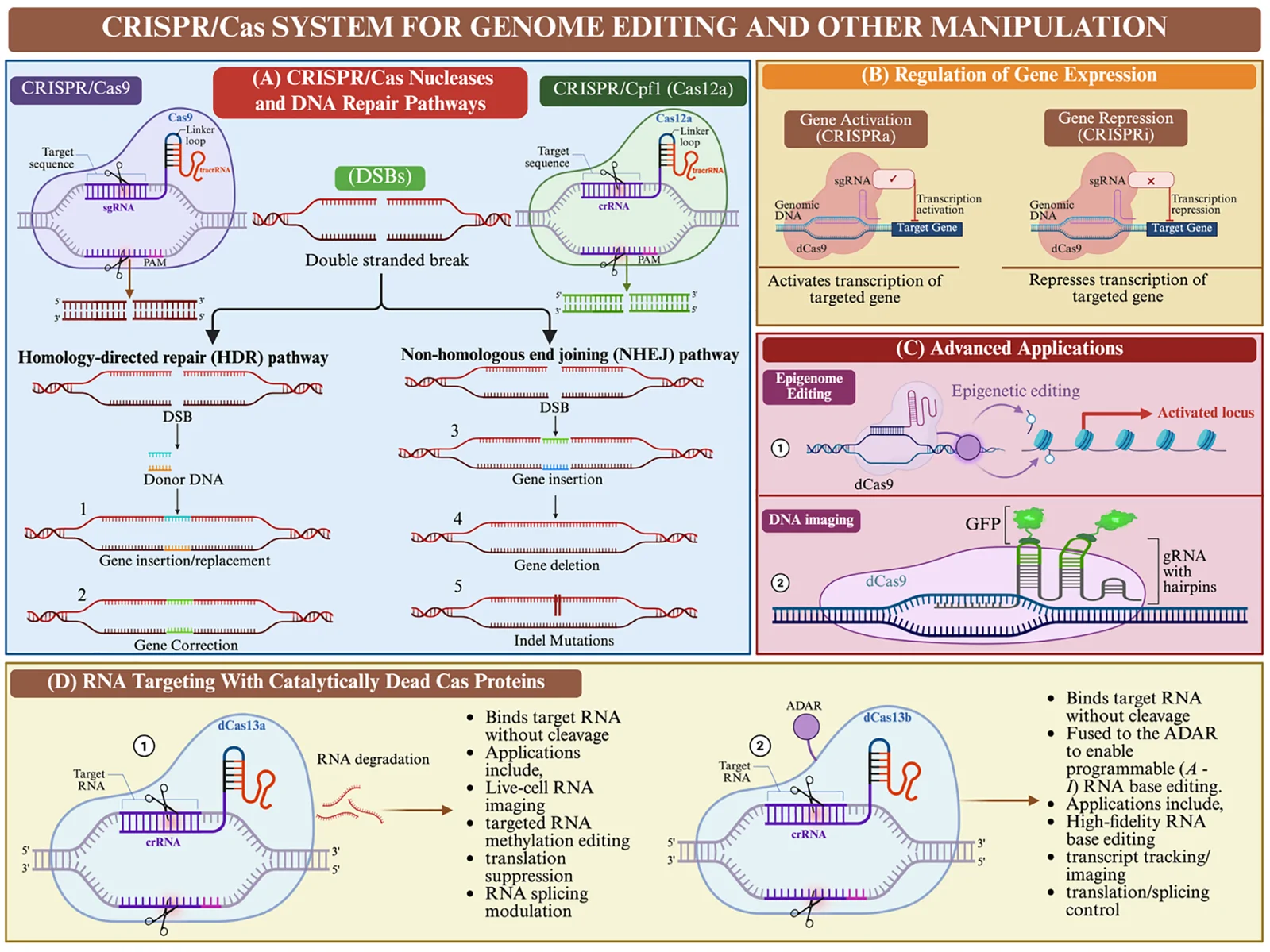
Structural architecture and diverse applications of the CRISPR-Cas toolset for plant genome engineering, transcriptional modulation, and RNA editing. A comprehensive schematic showcasing the adaptability of Class 2 CRISPR systems from standard DNA cleavage to catalytically inactive variations for epigenome, transcriptome, and epitranscriptome engineering. **(A)** CRISPR/Cas Nucleases and DNA Repair Pathways: Functional comparison between the monomeric SpCas9 and AsCpf1 (Cas12a) endonucleases. Cas9 recognizes a 3′ PAM and utilizes a sgRNA containing a structural linker loop to generate blunt-ended DSBs. In contrast, Cpf1 (Cas12a) recognizes a 5′ T-rich PAM, utilizes a single short crRNA with a distinctive 5′ stem-loop, and introduces staggered, sticky-ended DSBs with 4–5 nucleotide 5′ overhangs. Downstream repair of these DSBs proceeds via two main endogenous cellular pathways. HDR pathway: An error-free mechanism triggered in the presence of an exogenous donor DNA template, driving targeted transgene insertion/replacement (1) or precise allele substitution/gene correction (2). NHEJ pathway: An error-prone, template-independent repair mechanism that directly ligates broken DNA ends, frequently culminating in targeted gene insertion (3), gene deletion (4), or small insertion/deletion (indel) mutations (5) that disrupt reading frames. **(B)** Regulation of Gene Expression (CRISPRa and CRISPRi): Application of catalytically inactive “dead” Cas9 (dCas9), which retains DNA-binding specificity but lacks cleavage activity. In Gene Activation (CRISPRa), dCas9 is fused to transcriptional activators to recruit endogenous RNA polymerase machinery and upregulate target gene transcription. In Gene Repression (CRISPRi), dCas9 is fused to transcriptional repressors (or physically blocks the promoter region) to sterically hinder transcription and downregulate target gene expression. **(C)** Advanced Applications of dCas9 Scaffolds: (1) Epigenome Editing: Fusion of dCas9 with chromatin-modifying enzymes (e.g., histone acetyltransferases, methyltransferases, or demethylases) to alter localized epigenetic landscapes, inducing chromatin remodeling and activating or silencing specific genomic loci without modifying the primary DNA sequence. (2) DNA Imaging: Visual tracking of physical genomic loci within living cells using dCas9 molecules tagged with GFP or engineered with specialized gRNAs carrying structural hairpins that scaffold fluorescent aptamers. **(D)** RNA Targeting with Catalytically Dead Cas Proteins: Expansion of the CRISPR toolset to direct post-transcriptional manipulation via RNA-guided ssRNA binding proteins: (1) dCas13a Systems: Programmable binding of target transcripts using a crRNA guide without inducing cleavage. This platform enables targeted RNA degradation, live-cell RNA imaging, RNA methylation editing, translation suppression, and alternative splicing modulation. (2) dCas13b Systems: Precision epitranscriptome editing via the fusion of dCas13b to the catalytic domain of ADAR. This architecture enables high-fidelity, programmable RNA base editing (A-to-I conversion, read as G during translation), as well as transcript tracking and translation control.

Beyond the induction of double-strand breaks, CRISPR technology has evolved into a multifunctional platform capable of regulating gene expression, modifying epigenetic states and visualizing chromatin organization without altering DNA sequence (Li et al., 2017a). These applications primarily exploit catalytically inactive Cas proteins, particularly dead Cas9 (dCas9), which retains sequence-specific DNA-binding activity but lacks nuclease function (**Figure 2**) (Potlapalli et al., 2024). Fusion of dCas9 with transcriptional activators or repressors enables programmable regulation of endogenous gene expression (CRISPRa and CRISPRi). Activation domains such as *VP64, VPR* and *p65*, and repression domains including *SRDX* and *KRAB*, have been successfully employed to modulate plant gene expression, allowing simultaneous activation and repression of multiple loci involved in development, stress responses and agronomic traits (Dominguez et al., 2016; Lowder et al., 2015). Similarly, dCas9 fused with epigenetic modifiers, including DNA demethylases and histone-modifying enzymes, enables locus-specific epigenome editing without introducing permanent DNA mutations. For example, targeted DNA demethylation using dCas9-TET1 has successfully reactivated the *FWA* locus in Arabidopsis, demonstrating the potential of programmable epigenetic engineering in plants (Li et al., 2018a; Kungulovski and Jeltsch, 2016). CRISPR platforms have also facilitated multiplex genome editing through the simultaneous expression of multiple guide RNAs (McCarty et al., 2020). Polycistronic tRNA-gRNA systems, ribozyme-mediated processing and Cas12a-based crRNA maturation have enabled efficient editing of multiple genes in a single transformation event, greatly accelerating trait pyramiding and functional genomics studies (Li et al., 2019). Furthermore, fluorescently tagged dCas9 proteins have enabled live-cell imaging of chromosomal loci, providing valuable insights into chromatin dynamics and genome organization in plant cells (Li et al., 2017a). Together, these next-generation CRISPR platforms substantially extend genome editing beyond targeted mutagenesis and provide powerful tools for transcriptional regulation, epigenome engineering, functional genomics and precision crop improvement (Abushinova et al., 2024).

## 3 DSB-independent precision genome editing: base editing and prime editing platforms

**Figure 3:**
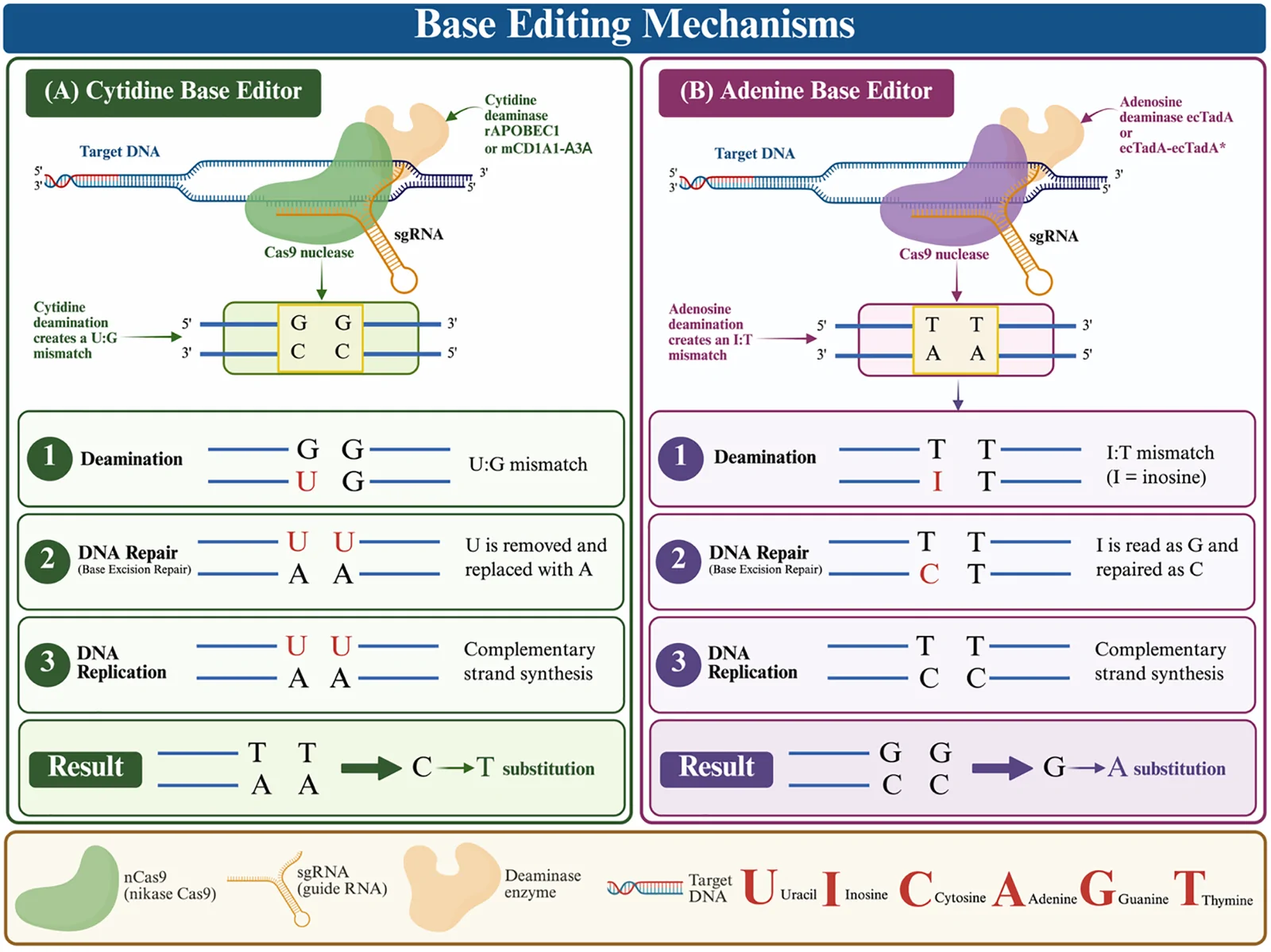
DNA single-nucleotide transitions via CBEs and ABEs. A step-by-step schematic illustrating the programmatic conversion of target base pairs without introducing DSBs, mediated by a nCas9, D10A variant complexed with a sgRNA and specialized deaminase enzymes. **(A)** CBE mechanism for C to T substitutions: Targeting and architecture, nCas9 scaffold targets the genomic locus via sgRNA homology, displaying a single-stranded DNA bubble within the editing window. A cytidine deaminase domain (such as rAPOBEC1 or human/monomorphic variants like MCD1A1-A3A) catalyzes the hydrolytic deamination of the target C to U on the non-target strand, producing a temporary U:G mismatch. Step 1 (Deamination): Hydrolytic conversion initiates the mismatch profile, altering the chemical composition of the exposed strand while nCas9 nicks the opposite, unedited strand to stimulate cellular mismatch repair. Step 2 (DNA Repair/Base Excision Repair): In typical cellular systems, the nicked G-containing strand is degraded and resynthesized using the U-containing strand as a template, or the cellular machinery incorporates the intermediate configuration depicted here where base conversion cascades through localized repair events. Step 3 & Result (DNA replication/complementary strand synthesis): During subsequent rounds of DNA replication, DNA polymerases recognize U as T and incorporate an A on the nascent complementary strand. This ultimately culminates in a permanent, clean C to T transition mutation (reversely tracked as a G to A substitution on the opposite strand) across the genomic locus. **(B)** ABE mechanism for A to G (and subsequent G to A) substitutions: Similar to the CBE, an engineered or evolved heterodimeric/monomeric bacterial adenosine deaminase (such as Escherichia coli TadA [ecTadA] or its evolved high-efficiency variant ecTadA-ecTadA*) is linked to nCas9. The complex converts a target A into I on the non-target single-stranded DNA bubble, yielding an initial I:T mismatch. Step 1 (Deamination): Hydrolytic deamination of adenine generates the inosine intermediate at the target nucleotide site. Step 2 (DNA Repair): The cellular repair machinery and high-fidelity DNA polymerases recognize I structurally as a G mimic. The nicked T-containing strand is preferentially repaired to incorporate a C opposite the inosine. Step 3 & Result (DNA replication/complementary strand synthesis): During replication and final complementary strand synthesis, the inosine-guided strand acts as a template for stable cytosine incorporation, which resolves into a permanent A to G transition (visualized on the target coding/non-coding sequence as a steady G to A substitution match depending on strand selection).

BE is a refined GE strategy that permits precise single base substitution without generating DSBs or requiring donor templates or homology-directed repair. This DSB-independent strategy provides a very efficient and predictable approach for engineering point mutations in plant genomes and has raised as a potential tool for hybrid crop betterment (**Figure 3**) (Chen et al., 2019).

The cytosine base editor (CBE) was the first BE system developed for targeted single-nucleotide modification. CBEs are comprised of a cytidine deaminase connected to a CRISPR-Cas9 nickase [nCas9 (D10A nCas9)] together with a uracil glycosylase inhibitor (UGI). This complex speed up the conversion of cytosine to uracil within a defined editing window at specific locus (Komor et al., 2016) (**Figure 3a**). During subsequent DNA replication or repair, the U is preferentially resolved as thymine, leading to permanent C-to-T (or G-to-A) substitution. The presence of UGI suppresses base excision repair by inhibiting uracil DNA glycosylase, thereby enhancing BE capability (Chen et al., 2025). One of the most widely used CBE architectures is base editor 3 (BE3), which incorporates the rat *APOBEC1* cytidine deaminase and has been applied successfully across multiple crop and animal species (Hess et al., 2017). Subsequent engineering efforts have produced improved CBE variants with enhanced specificity, editing efficiency, and expanded PAM compatibility. In addition to *APOBEC1*, several cytidine deaminase orthologs, including human APOBEC3A, human activation-induced deaminase (AID), and lamprey *PmCDA1*, have been connected to nCas9 to improve C-to-T conversion efficiency (Ma et al., 2016; Gehrke et al., 2018). Notably, *APOBEC3A*-based CBEs have been successfully applied in crops like potato, rice, and wheat, demonstrating high editing efficiencies (Zong et al., 2018).

Besides cytosine editing, adenine base editors (ABEs) were also created to enable targeted A-to-G (or T-to-C) conversions in genomic DNA (**Figure 3b**). ABEs are comprised of a derived transfer RNA adenosine deaminase (TadA) from *Escherichia coli* fused to nCas9 (Li et al., 2025). Through multi discs of directed evolution, highly efficient ABE variants were generated, with the seventh-generation ABE exhibiting robust and reliable editing performance (Kim and Kim, 2025). ABEs have since been widely applied in major crops, including rice and wheat, for precise gene optimization. Enhanced editing efficiencies of up to 60% have been reported in these crops through the combined use of optimized sgRNA architectures and multiple nuclear localization signals fused to nCas9 (Li et al., 2018b). BE permits many advantages over DSB-dependent GE approaches. First, it enables the introduction of precise nucleotide substitutions, including nonsense and missense mutations, without inducing chromosomal rearrangements such as large deletions or inversions. Second, BE generally exhibits higher efficiency and greater predictability than HDR-based methods (Chen et al., 2019). Third, the absence of DSBs reduces genome instability and unintended large-scale mutations. However, BE remains limited to few types of nucleotide substitutions and cannot efficiently mediate large insertions, gene replacements, or complex sequence modifications, which still require DSB-based strategies. Overall, BE technologies represent a valuable addition to the GE toolbox for agriculture, offering a precise and efficient means to generate genetic diversity and accelerate hybrid crop improvement (Chen et al., 2019).

Prime editing (PE) has emerged as a versatile DSB-free genome editing technology that enables precise genetic modifications without requiring donor DNA templates or relying on homology-directed repair (Lee et al., 2025). The system consists of a Cas9 nickase (nCas9) fused to an engineered reverse transcriptase (RT) and is guided by a prime editing guide RNA (pegRNA), which specifies both the target site and the desired genetic alteration (Mansi and Danai, 2026). Following target recognition, the Cas9 nickase introduces a single-strand nick, after which the RT copies the edit encoded within the pegRNA directly into the target DNA. Cellular DNA repair mechanisms subsequently incorporate the newly synthesized strand, enabling precise installation of targeted base substitutions, small insertions, and deletions while minimizing the generation of undesired insertions or deletions typically associated with DSB repair. Compared with conventional CRISPR/Cas9-mediated editing, PE substantially reduces the risk of large genomic rearrangements and off-target mutations arising from error-prone NHEJ (Chen et al., 2025). Moreover, unlike cytosine and adenine base editors, which are limited to specific transition mutations, PE can introduce all twelve possible base substitutions as well as small sequence insertions and deletions, making it one of the most versatile precision genome editing platforms developed to date (Chen et al., 2025). Although editing efficiencies in plants remain variable and are generally lower than those achieved with standard CRISPR/Cas9 systems, continuous improvements in pegRNA design, RT engineering, expression strategies, and optimized PE architectures (including *PE2, PE3, PE4*, and *PE5*) have substantially enhanced editing performance across diverse plant species (Al-Saadi et al., 2026; Lee et al., 2025). In plants, prime editing has been successfully demonstrated in major crops including rice, wheat, maize, tomato, and potato for the precise modification of genes associated with herbicide resistance, disease resistance, yield-related traits, and functional genomics. Li et al. (2025) expanded the utility of PE in rice by developing a nuclease-mediated prime editing (NM-PE) platform that exploits microhomology-mediated end joining for efficient *in situ* epitope tagging of endogenous genes. The authors further engineered PAM-flexible *ScCas9* and *SpRY*-based prime editors to broaden target accessibility and demonstrated simultaneous tagging of multiple endogenous genes, highlighting the potential of PE for functional genomics, protein localization studies, and precision crop improvement. Chen et al. (2025) demonstrated the application of PE in wheat by introducing the *P106S* amino acid substitution into the *TaEPSPS* gene to confer glyphosate resistance. The edited plants exhibited effective tolerance to field-recommended glyphosate doses while maintaining normal *TaEPSPS* expression and showing minimal fitness or yield penalties under non-stress conditions. These findings highlight the potential of PE to generate precise herbicide-resistant alleles for wheat breeding without compromising agronomic performance. Jiang et al. (2020) improved the efficiency of PE in maize by optimizing pegRNA expression. Using enhanced pegRNA expression strategies, the authors successfully introduced the *S621I* and *W542L* substitutions into the *ZmALS2* and *ZmALS1* genes with substantially higher editing efficiencies than those previously reported in rice. Their findings demonstrated that pegRNA optimization is an effective strategy for improving PE performance in plants and provided an important foundation for the further development of efficient plant prime editing systems. Wang et al. (2025) established a highly efficient protoplast-based transient transformation system for cotton that provides a rapid platform for functional genomics and genome editing studies. The system was successfully employed to validate PE vectors alongside applications such as subcellular localization and bimolecular fluorescence complementation assays, highlighting its value for accelerating the optimization and evaluation of precision genome editing technologies in cotton. Vu et al. (2024) addressed the limited efficiency of PE in dicot plants by developing an optimized PE system incorporating engineered pegRNAs, an RNA chaperone, a Pol II–Pol III composite promoter, and a viral replicon strategy. This approach markedly improved editing efficiency in *Arabidopsis* and tomato, enabled stable heritable transmission of edited alleles, and demonstrated high editing accuracy and multiplexing capability, thereby advancing the practical application of PE in dicot crop improvement. Veillet et al. (2020) provided one of the earliest demonstrations of PE in potato by precisely modifying the *StALS1* gene in a tetraploid and highly heterozygous genetic background. The study showed that PE could simultaneously introduce transition and transversion mutations, underscoring its potential for generating precise gain-of-function alleles and expanding precision breeding opportunities in clonally propagated crops. As delivery systems and editing efficiencies continue to improve, prime editing is expected to become an indispensable tool for precision crop improvement by enabling predictable and programmable genome modifications without introducing double-strand DNA breaks (**Table 1**). Consequently, this technology represents a significant step toward next-generation plant breeding and the development of improved crop varieties with enhanced agronomic performance (Wang et al., 2025).

**Table 1:** Comparative overview of CRISPR-based genome editing strategies in plants: mechanisms, advantages, limitations, efficiencies, and representative applications.

Editing strategy	Mechanism	DNA break requirement	Repair pathway	Typical editing outcome	Advantages	Limitations	Relative editing efficiency in plants	Representative plant applications/examples	Best suited applications
NHEJ-mediated CRISPR/Cas9 editing	Cas9 or Cas12 induces a targeted DSB that is repaired through the endogenous NHEJ pathway, frequently generating small insertions or deletions (indels).	DSB	NHEJ	Gene knockouts, frameshift mutations, small deletions, multiplex mutagenesis	Simple design, highly efficient, works in most plant species, suitable for multiplex editing, no donor template required	Repair outcomes are unpredictable; unsuitable for precise nucleotide substitutions or targeted gene insertion; potential off-target mutations and large genomic rearrangements	High	*OsSWEET* genes for bacterial blight resistance in rice; *MLO* knockout for powdery mildew resistance in wheat; *ARGOS8* editing in maize for drought tolerance; *SlCLV3* editing in tomato for increased fruit size	Functional genomics, trait knockout, disease resistance, crop domestication
HDR-mediated CRISPR editing	Cas-induced DSB is repaired using an exogenous homologous donor DNA template, allowing precise sequence replacement or targeted insertion.	DSB	HDR	Gene replacement, targeted gene insertion, allele replacement, promoter engineering	High editing precision; enables accurate gene insertion and allele replacement	Low efficiency in plants due to dominance of NHEJ, limited donor delivery, and cell-cycle dependence; technically demanding	Low	Targeted insertion of herbicide resistance genes in rice; precise promoter replacement in tomato; HDR-assisted trait engineering using geminivirus replicons	Gene knock-in, allele replacement, metabolic engineering
Base Editing (CBE)	nCas9 or dCas9 fused with cytidine deaminase converts C•G to T•A without introducing DSBs.	No DSB	No HDR required	C→T (G→A) substitutions	High precision; reduced indels; efficient in both monocots and dicots; suitable for SNP correction	Limited to transition mutations within a defined editing window; possible bystander edits	High	ALS gene editing for herbicide resistance in rice, wheat, tomato, watermelon and canola; improvement of disease resistance genes	Single nucleotide substitution, trait improvement, herbicide resistance
Base Editing (ABE)	nCas9 fused with adenine deaminase converts A•T to G•C without DSB formation.	No DSB	No HDR required	A→G (T→C) substitutions	Precise editing with minimal genomic disruption; lower indel frequency	Restricted to transition mutations; editing window constraints; PAM dependency	Moderate–High	Editing of *ALS, ACC, EPSPS* and yield-related genes in rice and wheat	Precise SNP introduction, functional genomics, breeding elite alleles
Prime Editing (PE)	Cas9 nickase fused to reverse transcriptase uses a prime editing guide RNA (pegRNA) encoding the desired edit, enabling direct DNA synthesis without donor DNA.	Single-strand nick (no DSB)	Reverse transcription-mediated repair	All 12 base substitutions, small insertions and deletions	Most versatile precision editing platform; no donor template required; reduced genomic instability; capable of multiple edit types	Editing efficiency remains lower than NHEJ; pegRNA design is complex; performance varies among plant species and genomic loci	Moderate (species dependent)	Jiang et al. (2020): improved PE efficiency in maize through optimized pegRNA expression; Veillet et al. (2020): precise editing of *StALS1* in potato; Vu et al. (2024): enhanced PE efficiency and heritable editing in tomato and *Arabidopsis*; Li et al. (2025): NM-PE for endogenous gene tagging in rice; Chen et al. (2025): glyphosate-resistant *TaEPSPS* wheat	Precision breeding, allele engineering, gain-of-function mutations, targeted sequence correction
CRISPR interference (CRISPRi)	dCas9 fused to transcriptional repressors blocks transcription without modifying DNA sequence.	No DNA break	Not applicable	Gene repression	Reversible, programmable, no permanent mutation	Requires continuous expression; incomplete repression possible	Moderate	Repression of *FWA*, miRNAs and developmental regulators in *Arabidopsis*	Functional genomics, regulatory network studies
CRISPR activation (CRISPRa)	dCas9 fused with transcriptional activators promotes transcription of endogenous genes.	No DNA break	Not applicable	Gene activation	Activates native genes without genome modification; multiplex activation possible	Variable activation efficiency; delivery of large constructs	Moderate	Activation of multiple developmental and stress-responsive genes in Arabidopsis and tomato	Trait regulation, metabolic engineering
Epigenome Editing	dCas9 fused with DNA methylases, demethylases or histone modifiers alters epigenetic marks at specific loci.	No DNA break	Not applicable	DNA methylation or histone modification	Reversible regulation; no DNA sequence alteration; useful for studying epigenetic inheritance	Long-term stability and heritability remain uncertain	Moderate	dCas9-TET1-mediated demethylation of the *FWA* locus in *Arabidopsis*; targeted histone modification studies	Epigenetic regulation, gene expression studies
Multiplex CRISPR Editing	Simultaneous expression of multiple gRNAs enables concurrent editing of several genomic loci.	Usually DSB (Cas9/Cas12)	Mainly NHEJ	Multiple gene knockouts or trait pyramiding	Accelerates breeding of polygenic traits; reduces breeding cycles	Variable efficiency among targets; increased design complexity; potential off-target accumulation	High	Simultaneous editing of yield, flowering and disease resistance genes in rice, maize and tomato using tRNA-gRNA and Cas12a systems	Trait pyramiding, *de novo* domestication, complex trait engineering

## 4 Plant transformation through CRISPR/Cas reagents

Efficient transmission of GE compounds into crop cells is a reproving step in CRISPR/Cas-mediated hybrid transformation. Successful GE depends not only on the design of the CRISPR components but also on the technique applied to introduce editing reagents into plant tissues and initiate the editing process (Chen et al., 2019). Many delivery strategies have been generated to transfer CRISPR/Cas reagents, like RNA, RNP and DNA complexes, into crop cells. Among these approaches, protoplast transfection is commonly utilized for transient expression studies and rapid evaluation of guide RNA efficiency. However, from protoplast whole plant regeneration still remains challenging in several crop species, limiting its routine application in hybrid breeding programs (Chen et al., 2025). In contrast, *Agrobacterium tumefaciens* and particle bombardment-based transformation are the most extensively utilized approaches for generating stable, genome-engineered crops (Hamada et al., 2018). Particle bombardment enables direct transmission of CRISPR/Cas DNA, RNA, or RNPs into crop cells, while *Agrobacterium*-mediated transformation facilitates efficient integration or transient expression of CRISPR constructs, particularly in dicot species (Chen et al., 2019). Together, these delivery systems provide flexible and effective platforms for introducing CRISPR/Cas reagents into diverse plant species, thereby allowing precise GE and accelerating the development of improved hybrid crops.

## 5 Hybrid plant genome modifications through CRISPR/Cas DNA

CRISPR/Cas DNA-based delivery remains the most extensively utilized approaches for crop genome modification. In this strategy, DNA cassettes encoding the Cas nuclease and sgRNA are delivered into crop cells, where they are expressed to induce targeted genome edits. The CRISPR/Cas components may integrate randomly into the plant genome, enabling stable expression, although this can also lead to sgRNA degradation or prolonged Cas activity (Kis et al., 2019).

### 5.1 Genome editing via CRISPR/Cas DNA stable expression

**Figure 4:**
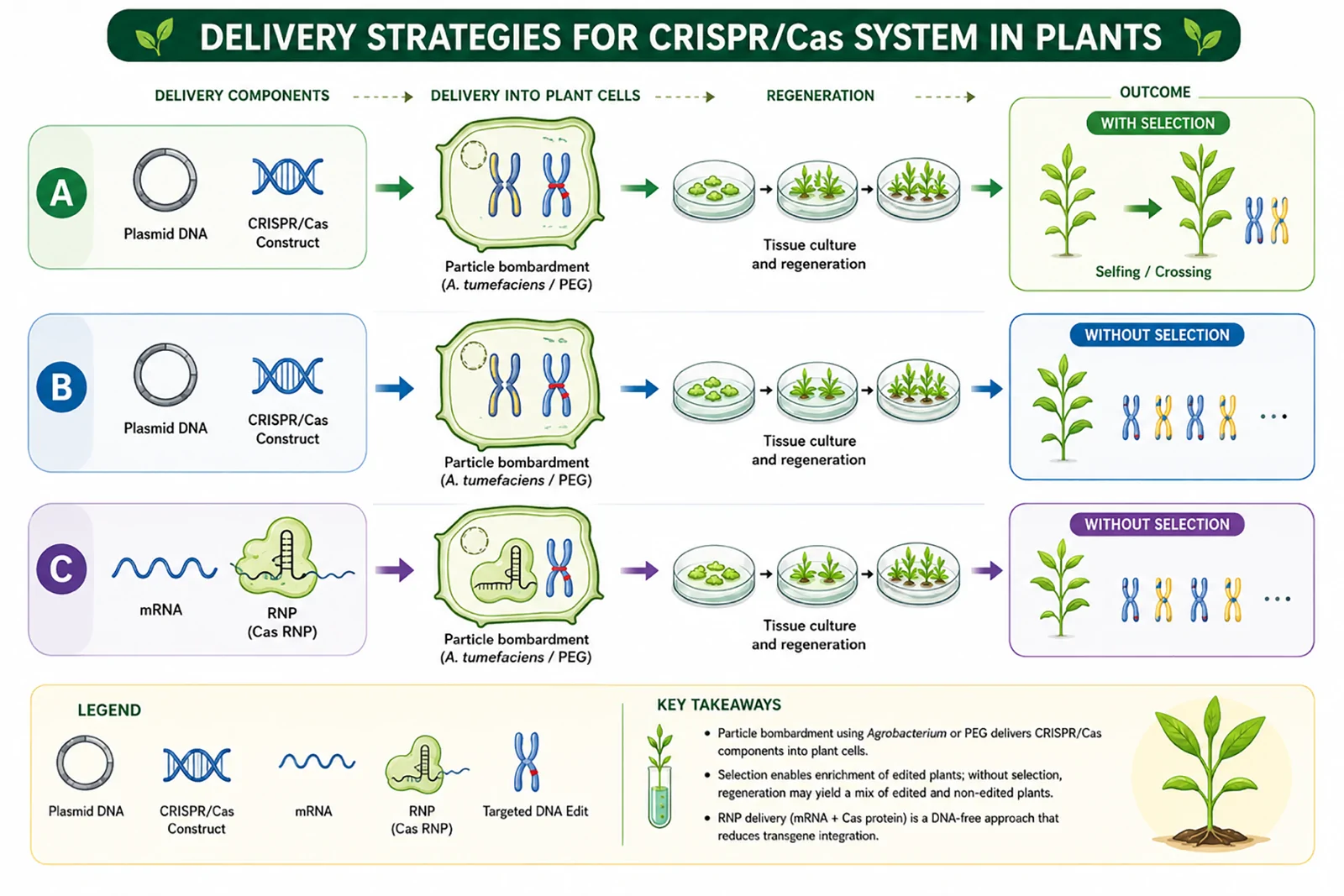
CRISPR/Cas delivery methods in plants. **(A)** CRISPR/Cas DNA combination with antibiotic or herbicide selection through traditional delivery methods. Genetic segregation through crossing and selfing help in obtaining transgene free plants. **(B, C)** transgene and DNA free genome editing transient delivery systems. Ribonucleo protein particles (RNPs), mRNA and CRISPR-DNA are CRISPR reagents. RNP, mRNA and CRISPR-DNA get degrade once transient expressed and edited plant can be re-developed with no selection pressure.

Stable GE is achieved through the transmission of recombinant CRISPR DNA into crop cells utilizing techniques like particle bombardment or *Agrobacterium tumefaciens*-mediated transformation (Shafique et al., 2022). Following delivery, the transgene integrates into the crop genome and is expressed under the control of specific promoters, enabling targeted modification of genes associated with desirable hybrid traits, including stress tolerance and yield components (**Figure 4a**). Despite its effectiveness, stable CRISPR/Cas DNA integration presents several limitations. Persistent expression of the Cas nuclease increases the likelihood of off-target mutations, which may restrict its acceptance in commercial hybrid breeding (Mansi and Danai, 2026). To mitigate these concerns, transgene-free edited crops could be created through genetic segregation through crossing or selfing edited lines, allowing removal of the CRISPR/Cas transgene in subsequent generations (Chen et al., 2019). Several strategies have been deployed to facilitate this process. For example, fluorescent marker cassettes have been employed to visually identify CRISPR/Cas-containing plants (Gao et al., 2017), while suicide genes such as *BARNASE* and *CMS2* have been used to selectively eliminate transgene-containing embryos or pollen in T_0_ plants (He et al., 2018). However, genetic segregation techniques are not applicable to crops which are propagated through vegetation like banana, cassava, and potato, where transgene removal through breeding is not feasible. Additionally, unintended integration of CRISPR/Cas DNA at non-target genomic locations remains a concern, further emphasizing the need for alternative transgene-free strategies (Potlapalli et al., 2024).

### 5.2 CRISPR/Cas DNA transient expression and genome modifications

Transient expression of CRISPR/Cas reagents offers an effective alternative for generating genome-edited crops without stable transgene integration (**Figure 4b**). In this approach, CRISPR/Cas components are introduced into plant cells through transient delivery methods, such as PEG-mediated transfection of protoplasts, particle bombardment, Agrobacterium-mediated transient transformation, viral vector-based delivery, or the direct introduction of preassembled CRISPR/Cas RNP complexes (Potlapalli et al., 2024). These components are expressed only temporarily, allowing targeted genome editing to occur before the introduced DNA, RNA, or protein is degraded, thereby minimizing stable transgene integration and facilitating the recovery of transgene-free edited plants (Basu et al., 2023). This strategy eliminates the need for antibiotic or herbicide selection, enabling regeneration of edited crops that do not carry foreign DNA sequences. Transient CRISPR/Cas-based GE was first demonstrated in wheat where CRISPR/Cas9 plasmids were transmitted via particle bombardment into immature embryos and regenerated without selection pressure (Zhong et al., 2018). Remarkably, up to 86.8% of the resulting T_0_ plants carried targeted mutations without detectable transgene integration. This approach significantly reduced tissue culture time and minimized regulatory concerns associated with transgenic crops. Transient delivery has also been successfully combined with BE systems, including cytosine and adenine base editors, to generate precise nucleotide substitutions without introducing foreign DNA in crop genome (Li et al., 2018b; Zong et al., 2018). Furthermore, biolistic delivery and protoplast transfection have been deployed to create transgene-free edited crops in potato (tetraploid) and tobacco (Andersson et al., 2017; Lin et al., 2018). In addition, transient *Agrobacterium*-based expression of CRISPR/Cas reagents have been reported in tobacco, further expanding the toolkit for transgene-free plant GE (Chen et al., 2018).

### 5.3 DNA-free genome modification through CRISPR/Cas ribonucleoproteins and *in-vitro* transcripts

DNA-based CRISPR/Cas techniques have adopted for plant GE and have enabled the creation of transgene-free crops through segregation strategies. However, complete elimination of foreign DNA remains challenging, as short or degraded DNA segments may yet integrate into crop genome (Ali et al., 2023). To overcome these limitations, RNA and RNP-based delivery techniques have been created as truly DNA-free GE strategies. Both *In-vitro* transcribed Cas9 mRNA and sgRNA have been transmitted in crop cells to induce genome edits without DNA integration. For example, Zhang et al. (2018a) introduced sgRNA and Cas9 transcripts into wheat immature embryos via particle bombardment, resulting in edited plants without detectable transgene integration (**Figure 4c**). Although they reported lower editing efficiency as compare to that achieved using DNA-based expression systems likely due to transient and unstable nature of RNA this approach substantially reduced off-targets effects and eliminated the foreign DNA insertion risk.

To further improve efficiency and precision, CRISPR/Cas RNP-based genetic engineering has now raised as a robust DNA-free alternative. In this system, preassembled Cas9 protein sgRNA complexes are transmitted directly in plant cells, bypassing the need for transcription and translation. RNP-mediated editing exhibits efficiencies comparable to plasmid-based systems while significantly reducing off-target activity, as the complexes degrade rapidly after cleavage (Liang et al., 2017; Woo et al., 2015; Svitashev et al., 2016). Woo et al. (2015) first demonstrated CRISPR/Cas9 RNP-based genome modifications in plant protoplast of lettuce, tobacco, *Arabidopsis thaliana*, and rice utilizing polyethylene glycol-based transfection. Recreated lettuce mutants showed up to 46% mutation frequencies, with no detectable off-target mutations in lettuce and *Arabidopsis*. Subsequently, RNP-mediated editing has been successfully reported in protoplasts of potato, petunia, apple, and grape (Malnoy et al., 2016; Subburaj et al., 2016). In addition, Kim et al. (2017) demonstrated the application of *AsCpf1* and *LbCpf1* RNPs in tobacco and soybean, further expanding the CRISPR toolbox for DNA-free editing. Gogoi et al. (2025) also revealed the combined application of DNA free methods and nanobiotechnology offers solutions to overcome conventional GE techniques.

Despite these advances, efficient plant regeneration from protoplasts particularly in monocot crops remains a major bottleneck. Consequently, particle bombardment has become the preferred method for RNP delivery in cereals. RNP-mediated GE via biolistic has successfully evidenced in maize and wheat (Poddar et al., 2023; Liang et al., 2018). Svitashev et al. (2016) transmitted preassembled Cas9/sgRNA RNPs in embryos of *Zea mays* without selection, gaining 2.4% to 9.7% editing efficiencies with approximately 10% of mutants exhibiting biallelic modifications and no detectable off-target effects. Similarly, Liang et al. (2017) reported high on-target efficiency and low off-target activity in wheat embryos edited using RNP delivery, comparable to transient DNA-based approaches. Given the successful application of RNPs for high-fidelity BE in animal systems (Kocsisova and Coneva, 2023). RNP-mediated BE represents a promising future direction for precise and DNA-free genome modification in plants. Such approaches hold significant potential for accelerating hybrid crop improvement while minimizing regulatory and biosafety concerns.

## 6 Plant breeding and precision applications of CRISPR/Cas

### 6.1 Trait improvement through gene knockout

Several strategies have been developed to improve hybrid crops, but elimination of negative regulatory genes remains one of the most effective approaches. Targeted gene knockout using CRISPR/Cas9 enables suppression of genes that negatively regulate desirable traits, thereby improving crop performance (Gao et al., 2017). Among GE tools, CRISPR offers a simple, efficient, and versatile platform for gene knockout and has applied in plant breeding. To date, CRISPR-based knockouts have been utilized to boost multiple agronomic traits, including tolerance to abiotic and biotic stressors, yield-related traits, and crop quality. This approach has also enhanced hybrid-breeding efficiency by accelerating the creation of elite parental lines (Mohamed et al., 2024).

### 6.2 Yield improvement

Plant yield is a primary breeding objective to ensure food security. Yield is considered as a complex quantitative trait influenced by multi genetics and environmental factors, including water availability, soil fertility, disease pressure, and climate. Targeted disruption of genes acting as negative regulators of yield-liked characters has proven effective in improving productivity. For example, knockout of *OsGn1a* increases grain number per plant, while disruption of *OsAAP3* enhances tiller number. Similarly, mutations in *DEP1* (*OsDEP1* and *TaDEP1*) improve panicle architecture, and knockouts of *TaGASR7*, *OsGLW2*, *OsGW5*, and *TaGW2* increase grain weight in rice and wheat (Li et al., 2016a, b; Liu et al., 2017; Lu et al., 2018a). Concurrent knockout of multiple grain weight associated genes (*TGW6, GW5*, and *GW2*) in rice resulted in trait pyramiding and a substantial enhancement in grain weight (Xu et al., 2016; Zeb et al., 2025). Since most yield-related traits are controlled by QTLs, single-gene knockouts may not always produce large yield gains. To address this limitation, Huang et al. (2018) combined CRISPR editing with whole-genome sequencing and pedigree analysis to identify and modify multiple yield-associated genes in rice. By targeting 57 candidate genes identified from elite cultivars and the Green Revolution variety IR8, this study offered important insights underlying genetics associated with biomass accumulation and demonstrated a scalable strategy for molecular breeding of high-yielding hybrids.

### 6.3 Quality improvement

GE has also been extensively utilized in boosting quality traits in hybrid crops, including nutritional value, starch composition, fragrance, shelf life, and processing quality. *Waxy* gene knockout in rice reduces amylose content, resulting in improved cooking and eating quality (Fu et al., 2023; Zhang et al., 2018a). Similar modifications in maize have facilitated the development of waxy hybrids for commercial use (Luo et al., 2024). Mutations in *SBEIIb* have enabled the production of high-amylose and resistant starch varieties, offering potential health benefits for individuals with chronic diseases related to diet (Sun et al., 2017). Fragrance is another economically valuable trait in rice. Mutation of *BADH2* leads to accumulation of 2-acetyl-1-pyrroline, the primary aromatic amalgam in fragrant rice. GE of *OsBADH2* has generated rice lines with significantly higher fragrance levels than naturally occurring aromatic varieties (Shan et al., 2015). CRISPR/Cas technology is particularly valuable for editing large gene families. For example, *α-gliadin* genes in wheat encode gluten proteins that trigger celiac disease in susceptible individuals. Simultaneous targeting of conserved domains within this gene family resulted in wheat genotypes having low gluten, demonstrating the feasibility of producing low-gluten hybrids (Sánchez-León et al., 2018). Additional quality enhancements achieved through CRISPR/Cas editing include enhanced oleic acid percentage in *False Flax* and *Brassica napus* (Jiang et al., 2017; Okuzaki et al., 2018), extended shelf life in tomato (Ito et al., 2015; Li et al., 2018c), increased γ-aminobutyric acid and lycopene levels in tomato (Nonaka et al., 2017), and reduced toxic glycoalkaloids in *Solanum tuberosum* (Nakayasu et al., 2018).

### 6.4 Enhancing tolerance to biological and environmental factors

Biotic and abiotic factors are major hurdles in reducing crop yield and quality. CRISPR/Cas-mediated knockouts have been widely applied to boost resistance to pathogens, insects, and environmental stresses in hybrid crops. Knockout of MLO associated genes in tomato and wheat has conferred resistance to powdery mildew (Nekrasov et al., 2017; Wang et al., 2014). In rice, disruption of *OsERF922* improved resistance to blast disease, while promoter editing of *OsSWEET13* reduced susceptibility to bacterial blight disease Resulting from *Xanthomonas oryzae* (Zhou et al., 2015; Wang et al., 2016). Virus tolerance has also been achieved through editing host susceptibility genes, like *eIF4E* in cucumber and *eIF4G* in rice (Chandrasekaran et al., 2016; Macovei et al., 2018), as well as CLCuD in cotton (Shafique et al., 2022). CRISPR editing has further enabled tolerance to insect pests. Knockout of *OsCYP71A1* disrupted serotonin biosynthesis and boosted silicon percentages in rice, enhancing tolerance to stem borers and planthoppers (Lu et al., 2018b). Heavy metal stress poses a serious peril to crop yield and food safety. Targeted disruption of metal transporter genes such as *OsHAK1, OsNramp5*, and *OsARM1* in rice reduced the accumulation of arsenic and cadmium, improving crop safety without compromising yield (Haider et al., 2023; Nieves-Cordones et al., 2017; Wang et al., 2017).

### 6.5 Acceleration of hybrid breeding through CRISPR/Cas

Hybrid breeding plays a central role in modern crop improvement by boosting productivity and stabilizing performance under diverse conditions. The creation of reliable male-sterile lines is a prerequisite for efficient hybrid seed production. Traditional approaches to induce male sterility are time-consuming and often unstable, whereas CRISPR/Cas-based GE has enabled rapid and precise generation of male-sterile lines across multiple crops. CRISPR/Cas has also effectively utilized to produce high-quality male-sterile lines by targeting key fertility-related genes, including *TMS5* in rice and maize (Zhou et al., 2016; Li et al., 2017b), *MS45* in wheat (Singh et al., 2018), and *CSA* in rice (Li et al., 2016c). These advances have significantly streamlined hybrid seed production and improved breeding efficiency. Reproductive barriers remain a major challenge in exploiting heterosis, particularly in inter-subspecific hybrids such as *indica–japonica* rice. GE has enabled precise manipulation of hybrid sterility loci to tackle these hurdles. For example, at the S1 locus, disruption of *OgTPR1* and SaM/SaF at the Sa sterility locus restored fertility in *indica–japonica* hybrids (Xie et al., 2017a, b). Similarly, Shen et al. (2017) demonstrated that Sc-I allele few copies knockout significantly improved *indica–japonica* male fertility. Yu et al. (2018) further reported that editing *ORF2*, a toxin gene involved in a “suicide gene” mechanism, enhanced hybrid fertility between these subspecies. Beyond fertility restoration, GE has enabled transformative advances in hybrid breeding by fixing heterozygosity through clonal seed propagation. Knockout of key meiotic genes (*OSD1, PAIR1*, and *REC8*) in rice replaced meiosis with mitosis, generating unreduced gametes. Subsequent studies demonstrated that mutation of *MTL* (MATRILINEAL) (Wang et al., 2019) or egg-cell-specific activation of *BBM1* (Khanday et al., 2019) enabled asexual seed formation, effectively preserving hybrid Vigor across generations. In addition to fertility manipulation, CRISPR/Cas has accelerated hybrid breeding by enabling rapid modification of other breeding-relevant traits. These include shortening the growth cycle (Li et al., 2017c), boosting haploid induction efficiency (Yao et al., 2018; Dong et al., 2018), overcoming self-incompatibility in crops such as potato (Ye et al., 2018), and increasing silique shatter tolerance in oilseed crops (Braatz et al., 2017). Collectively, these applications highlight the power of CRISPR to dramatically reduce breeding cycles and reshape modern hybrid crop improvement.

## 7 Hybrid crop trait improvement through knock-in and gene replacement

Advances in GE technologies, together with the availability of superior crop genome sequences, have greatly expanded opportunities to introduce desirable traits into hybrid crops. Early GE approaches like ZFNs and TALENs enabled precise targeting of specific genomic loci. However, these approaches are labor-intensive, costly, and require complex protein engineering, limiting their broader adoption (Sharma et al., 2025). Conversely, the CRISPR provides a simpler, highly effective, and economical platform for precise genome modification and has rapidly become the preferred tool for functional genomics and crop betterment. CRISPR has also been widely employed to improve agronomically important traits like disease resistance, nutrient use efficiency, stress tolerance, and yield. While many traits can be enhanced through gene knockout, knock-in and gene replacement strategies enable more precise manipulation, particularly when enhanced gene expression or allele substitution is required (Chen et al., 2019). A notable example is the introduction of drought-resistant maize, wheat, rice, tomato, cotton and pulses through CRISPR-based gene replacement. Shi et al. (2017) enhanced drought tolerance through substituting the native promoter of *ARGOS8* with the stronger promoter *GOS2* using HDR. In maize*, ARGOS8* encodes a suppressor of ethylene signaling and exhibits low basal expression. Increased expression of *ARGOS8* reduced ethylene sensitivity, resulting in improved drought tolerance without yield penalty (Ali et al., 2026). This study highlighted the power of promoter replacement as an alternative to gene knockout for trait improvement. Similarly, knockout of three *TaMLO* homologs in wheat conferred durable immunity against powdery mildew (Yu et al., 2017).

HDR efficiency in plants remains a major challenge; however, geminivirus-based replicon systems have been developed to increase donor template copy number and enhance gene targeting efficiency. Such systems have been adequately applied in *Solanum tuberosum* (Butler et al., 2016), *Solanum lycopersicum* (Čermák et al., 2015; Dahan-Meir et al., 2018), *Oryza sativa* (Wang et al., 2017b), *Triticum aestivum* (Gil-Humanes et al., 2017), and *Manihot esculenta* (Hummel et al., 2018). For instance, Čermák et al. (2015) introduced the *CaMV 35S* upstream promoter of the *ANT1* gene in *Solanum lycopersicum*, resulting in constitutive expression and accumulation of anthocyanins, producing purple-colored fruits with enhanced nutritional value. In addition, CRISPR/Cas9-based editing has been also introduced to develop resistance to geminiviruses like tomato yellow leaf curl virus and CLCV virus through targeting viral genomes or host susceptibility factors (Ali et al., 2016; Dahan-Meir et al., 2018). Precise nucleotide substitution through HDR has also been exploited to generate herbicide-resistant crops. Genes expressing *acetolactate synthase (ALS)* and *5-enolpyruvylshikimate-3-phosphate synthase (EPSPS)* are prime targets for herbicide tolerance. Substitution of key amino acids within conserved domains of these enzymes confers resistance to glyphosate or sulfonylurea-based herbicides. Such modifications have been successfully introduced into *Glycine max* (Li et al., 2015a), *Zea mays* (Kaul et al., 2024), and *Oryza sativa* (Li et al., 2025). Beyond stress tolerance and herbicide resistance, CRISPR-based knock-in strategies have been utilized to improve nutritional quality. Potato, an important staple crop, contains wild relatives with superior nutritional traits; however, conventional breeding is slow due to sexual incompatibility. GE has enabled rapid introduction of beneficial alleles into elite potato lines, improving nutritional value and yield within a short timeframe (Li et al., 2016d). Although small insertions or deletions may occur at recombination junctions during HDR, these modifications generally do not affect gene function and shown the efficacy of precise gene replacement in crop plants (Fu et al., 2023).

## 8 Hybrid plants and applications of base editors

Several agronomically essential characteristics are determined by SNPs located in coding or non-coding genome portions. BE, which allows precise nucleotide replacements without inducing DSBs, therefore represents a powerful approach for crop improvement and hybrid breeding (Shafique et al., 2022). One of the most successful applications of BE in crops is the introduction of herbicide tolerance. Editing of *ALS* gene has developed resistance to imidazolinone or sulfonylurea herbicides in *Oryza sativa* (Shimatani et al., 2017), *Triticum aestivum* (Zong et al., 2018), *Arabidopsis* (Chen et al., 2017), and *Citrullus lanatus* (Tian et al., 2018). In rice, ABEs editing of the *acetyl-CoA carboxylase* (*ACCase*) conferred tolerance to *haloxyfop-R-methyl*, further demonstrating the precision and utility of base editors for trait improvement (Li et al., 2018b). BE has also been applied to modulate RNA splicing, an important regulatory pathway that permits a single gene to create several protein isoforms. In eukaryotes, splicing is governed by conserved donor (GU) and acceptor (AG) sites at intron boundaries. Targeted base substitutions within these conserved motifs can disrupt or alter splicing patterns. For example, BE of splice acceptor sites altered *AtPDS* mRNA splicing in Arabidopsis (Kang et al., 2018), while replacement of guanine to adenine at a splice donor site caused intron retention in *AtHAB1*, resulting in hypersensitivity to abscisic acid (Xue et al., 2018). Likewise, Li et al. (2019) induced *AtMTA* mis-splicing and generated dual mutants of *OsNAL1* and *OsGL1* in *Oryza sativa*, highlighting the dominance of BE to fine-tune gene expression without disrupting coding sequences.

## 9 Fine-tuning gene regulation for hybrid breeding

Precise regulation of gene expression, rather than alteration of coding sequences, is often sufficient to improve agronomic traits and accelerate hybrid breeding. Gene expression can be regulated at transcriptional, post-transcriptional, translational, and post-translational levels, all of which are governed by cis-regulatory elements that could possibly be targeted through GE. At the transcriptional level, modification of promoter regions through deletion or replacement of cis-regulatory elements has proven effective. Rodríguez-Leal et al. (2017) engineered promoter portions of quantitative trait genes (*SISP, SIS*, and *SICLV3*) to generate a spectrum of expression levels, resulting in improved yield-related traits. Such promoter engineering strategies allow breeders to fine-tune gene expression rather than completely abolish gene function. Translational regulation can also be manipulated through GE. Upstream open reading frames (uORFs) are prevalent cis-regulatory elements that typically inhibit translation of the main downstream coding region. It is estimated that nearly 35% of Arabidopsis transcripts contain one or more uORF (Von Arnim et al., 2014). Targeted deletion of uORF start codons using CRISPR/Cas9 increased translation efficiency of primary open reading frames (Zhang et al., 2018b). Editing of the uORF in *LsGGP2* significantly enhanced ascorbate content by 80–140%, demonstrating the value of translational control in crop quality improvement. In addition, regulatory elements located in untranslated regions (UTRs), including ribosome sites of entry and RNA secondary structures in the 5′ leader sequence, as well as elements in the 3′ UTR, play key roles in genetic expression regulation and represent promising targets for specific GE (Liang et al., 2016; Li et al., 2015b).

## 10 CRISPR-based antiviral strategies in hybrid crops

Viral diseases account for nearly 50% of plant disease-related yield losses worldwide (Zaidi et al., 2016). CRISPR/Cas systems provide a programmable antiviral defense mechanism by rightly targeting viral genetics elements. DNA viruses, like geminiviruses, require a dsDNA for replication. Consistent expression of Cas9 and virus-specific sgRNAs has been shown to strop viral replication through introducing mutations into essential viral regions (Ji et al., 2015; Ali et al., 2015). However, repair of CRISPR-induced breaks by the NHEJ pathway can generate viral escape mutants (Mehta et al., 2018). Targeting highly conserved viral sequences, such as the intergenic stem-loop region essential for replication, has proven effective in minimizing viral escape (Ali et al., 2016). Moreover, the utilization of viral promoters to transiently enhance Cas9 expression can further reduce off-target effects (Ji et al., 2018). RNA viruses generally pose a greater threat to crop yield than DNA viruses. PAM-independent RNA-targeting Cas proteins, like FnCas9 and Cas13 (formerly C2c2), have demonstrated strong antiviral activity against RNA viruses. FnCas9 suppressed replication of cucumber and tobacco mosaic virus (Zhang et al., 2018c), while Cas13-mediated RNA cleavage inhibited turnip mosaic virus replication (Abudayyeh et al., 2016).

## 11 Applications of CRISPR-generated plant mutant libraries

Genome-wide mutant libraries are invaluable resources for functional genomics and trait discovery. Traditional mutagenesis techniques, for examples EMS induced mutations, T-DNA-mediated insertion, and irradiation, generate random mutations and require multiple generations to establish genotype-phenotype relationships, making them time-consuming and labor-intensive. CRISPR technique has allowed the quickest creation of large-scale, targeted mutant libraries in plants. Two independent studies generated genome-wide knockout libraries in rice. Meng et al. (2017) targeted around 13,000 genes expressed in shoot tissues and produced nearly 14,000 independent T_0_ lines, while Lu et al. (2018c) targeted over 34,000 genes and generated approximately 90,000 transgenic plants, achieving homozygous mutants within a single generation. The use of sgRNA barcoding allowed direct linkage of genotype and phenotype, greatly accelerating functional analysis. Similarly, Jacobs et al. (2017) created a CRISPR-based mutant library targeting 54 immunity-linked leucine-rich repeated genes in *Solanum lycopersicum*. The presence of well-distributed, high-coverage mutant libraries provides a powerful platform for novel germplasm development and precision improvement of hybrid crop traits.

## 12 Future aspects

The rapid advancement of CRISPR–Cas GE has established it as a cornerstone technology for precision hybrid breeding and crop improvement. Future progress will increasingly depend on integrating CRISPR-based GE with plant synthetic biology to design programmable genetic circuits capable of fine-tuning complex traits including productivity, stress tolerance, and metabolic efficiency. Precise manipulation of promoters, enhancers, epigenetic marks, and synthetic regulatory elements using dCas9-based systems will enable controlled modulation of gene expression without altering coding sequences. GE also offers unprecedented opportunities to accelerate the domestication of wild and semi-domesticated plant species. By targeting domestication-related genes, CRISPR–Cas systems can rapidly introduce agronomically desirable traits while preserving genetic diversity, thereby expanding germplasm resources for hybrid breeding and boosting crop tolerance to changing climatic conditions.

Despite these advances, efficient delivery of GE components remains a critical bottleneck. Future research should prioritize the development of genotype-independent, tissue-culture-free delivery systems, alongside improved Agrobacterium-mediated transformation and novel physical or nanoparticle-based delivery strategies. Such improvements will broaden the applicability of GE across diverse crop species and elite hybrid lines. Enhancing editing precision will remain a major focus. Although off-target effects in plants are generally low, the adoption of high-fidelity Cas variants, optimized sgRNA design, RNP-based delivery, and advanced BE platforms will further improve specificity and regulatory acceptance. In parallel, overcoming the lower efficiency of HDR through manipulation of DNA repair pathways and improved donor template delivery will be essential for precise gene replacement and allele introgression. In addition to Agrobacterium-mediated transformation, other delivery systems are expected to play an increasingly important role in expanding the applicability of CRISPR technologies across diverse plant species. Rhizobium-mediated transformation, including systems based on *Rhizobium radiobacter* (formerly *Agrobacterium tumefaciens*) and *Rhizobium rhizogenes* (formerly *Agrobacterium rhizogenes*), remains one of the most reliable approaches for introducing genome-editing reagents into plant cells. While *R. radiobacter* is widely employed for stable genetic transformation in numerous dicotyledonous crops, *R. rhizogenes*-mediated hairy root transformation provides a rapid and efficient platform for functional genomics, root biology, metabolic engineering, and the validation of CRISPR/Cas constructs before stable transformation. Continuous improvements in bacterial strains, binary vector systems, and tissue culture protocols are expected to enhance transformation efficiencies and broaden the applicability of Rhizobium-mediated genome editing to recalcitrant crop species. Biolistic transformation (particle bombardment) also remains a valuable delivery strategy, particularly for cereals and other species that exhibit low susceptibility to bacterial transformation. This method enables the direct delivery of plasmid DNA, mRNA, or preassembled CRISPR/Cas RNP complexes into plant cells without relying on biological vectors. The use of RNPs through particle bombardment is especially attractive because it enables transient genome editing while minimizing stable transgene integration and reducing regulatory concerns associated with genetically modified crops. Furthermore, biolistic delivery has proven effective for plastid genome engineering, multiplex genome editing, and transformation of elite cultivars that are difficult to modify using conventional Agrobacterium-based approaches. Future optimization of microprojectile composition, delivery parameters, and tissue regeneration protocols is expected to further improve editing efficiency while reducing tissue damage and random DNA integration. Together with nanoparticle-assisted delivery, viral vectors, and emerging in planta transformation strategies, these delivery platforms are expected to overcome current genotype dependence and tissue culture limitations, thereby facilitating efficient, transgene-free, and genotype-independent genome editing for next-generation crop improvement. Finally, emerging technologies such as CRISPR-based gene drives hold potential for controlling invasive agricultural pests and weeds, but their ecological and ethical implications necessitate cautious evaluation. Until robust regulatory frameworks and risk-mitigation strategies are established, such applications should remain confined to controlled research settings. In summary, continued innovation in CRISPR–Cas technologies, coupled with responsible deployment and regulatory oversight, will be instrumental in defining the future trajectory of hybrid breeding, global food security and sustainable agriculture.

The rapid evolution of CRISPR technologies is shifting plant genome engineering from simple gene disruption toward precise, programmable, and predictable genome modification. Among the most promising advances, prime editing is expected to play a transformative role in future crop improvement because it enables all types of base substitutions as well as small insertions and deletions without generating DBS or requiring donor repair templates. Although its editing efficiency in plants remains lower than that of conventional CRISPR/Cas9 systems, continued optimization of pegRNAs, engineered reverse transcriptases, PAM-flexible nucleases, and delivery strategies is steadily improving its performance. These developments are anticipated to facilitate precise allele replacement and the rapid introduction of elite agronomic traits while minimizing unintended genomic alterations. Another emerging trend is multiplex genome editing, which enables the simultaneous modification of multiple genes using several guide RNAs within a single transformation event. Because many agronomic traits, including yield potential, abiotic stress tolerance, disease resistance, nutrient-use efficiency, and flowering time, are governed by complex polygenic networks, multiplex editing provides an efficient strategy for pyramiding beneficial alleles and accelerating precision breeding. Advances in polycistronic guide RNA expression systems, tRNA-processing platforms, Cas12a-mediated crRNA maturation, and programmable CRISPR arrays are expected to further improve multiplex editing efficiency while reducing breeding cycles and minimizing linkage drag. Beyond permanent DNA sequence modification, epigenome engineering is emerging as a powerful approach for regulating gene expression without altering genomic sequences. Catalytically inactive Cas proteins (dCas9 or dCas12) fused with DNA methyltransferases, DNA demethylases, histone acetyltransferases, histone deacetylases, or other chromatin-modifying enzymes enable locus-specific epigenetic remodeling. This technology provides opportunities to reversibly regulate genes controlling stress responses, flowering, developmental processes, and secondary metabolism while preserving the native DNA sequence. As knowledge of plant epigenetic inheritance expands, CRISPR-based epigenome editing may become an important tool for engineering environmentally responsive traits and studying transgenerational epigenetic regulation.

Recent advances in artificial intelligence (AI) and machine learning are also transforming CRISPR experimental design. AI-assisted computational models are increasingly being used to predict guide RNA efficiency, evaluate off-target activity, optimize PAM selection, design pegRNAs and base-editing guides, and identify optimal genomic targets across diverse plant genomes. The integration of large genomic datasets with predictive algorithms enables more accurate guide RNA design, reducing experimental costs while improving editing efficiency and specificity. Future AI-driven platforms are expected to automate many aspects of CRISPR experiment planning, facilitating rapid implementation of precision genome editing in both model plants and crop species. Continued innovation in delivery technologies will be equally critical for realizing the full potential of next-generation genome editing. Although Agrobacterium-mediated transformation remains the most widely adopted approach, emerging delivery platforms, including nanoparticle-mediated delivery, viral vectors, biolistic introduction of CRISPR RNPs, and genotype-independent in planta transformation systems, offer promising alternatives for overcoming species- and genotype-dependent transformation barriers. These approaches reduce reliance on tissue culture, minimize stable transgene integration, and improve the production of transgene-free edited plants. Future efforts integrating advanced delivery systems with prime editing, multiplex editing, and AI-assisted design are expected to significantly accelerate precision breeding and expand genome editing to previously recalcitrant crop species.

Although CRISPR/Cas-mediated genome editing has revolutionized targeted gene disruption, the efficient and precise insertion of exogenous DNA sequences (knock-in) remains one of the most significant challenges in plant genome engineering. Unlike gene knockouts, which primarily rely on the highly efficient NHEJ pathway, targeted DNA insertion requires the accurate integration of donor DNA at predefined genomic loci. Conventional knock-in approaches largely depend on HDR, in which a donor template carrying the desired genetic sequence is flanked by homologous arms that guide precise recombination following CRISPR-induced DNA cleavage. However, HDR-mediated knock-in remains inefficient in most plant species because of the predominance of NHEJ, limited availability of donor templates at the break site, genotype-dependent transformation efficiencies, and the restriction of HDR to the S and G2 phases of the cell cycle. Consequently, improving targeted DNA insertion efficiency has become a major objective in next-generation plant genome engineering. Recent advances have focused on the development of alternative knock-in strategies that overcome the limitations of classical HDR. These include homology-independent targeted integration (HITI), microhomology-mediated end joining (MMEJ)-based insertion, homology-mediated end joining (HMEJ), and CRISPR-associated transposase systems, which exploit endogenous DNA repair pathways to facilitate targeted sequence integration with reduced dependence on homologous recombination. In parallel, optimized donor construct design has emerged as a critical determinant of knock-in efficiency. Factors such as donor template configuration, homology arm length, donor topology, protection of donor DNA from degradation, and synchronized delivery of CRISPR components with repair templates have all been shown to substantially influence targeted insertion frequencies. Viral replicon systems, chemically modified donor templates, and strategies that physically tether donor DNA to CRISPR/Cas complexes have further improved donor availability at target loci, thereby enhancing precise DNA integration in several plant species. Future developments are expected to integrate advanced donor construct engineering with next-generation genome editing technologies, including prime editing, programmable recombinases, and CRISPR-associated transposons, to achieve efficient, predictable, and transgene-free targeted DNA insertion. Continued improvements in delivery systems, tissue culture-independent transformation methods, and AI-assisted donor construct optimization are likely to further increase knock-in efficiency across both model plants and elite crop varieties. These advances will facilitate precise gene replacement, targeted stacking of agronomically important genes, promoter engineering, metabolic pathway reconstruction, and trait pyramiding, thereby expanding the scope of precision breeding beyond gene disruption toward sophisticated genome engineering.

## 13 Conclusion

The unparalleled precision in generating targeted, sequence-specific, and genome-wide genetic variation by GE has transformed functional genomics and plant breeding. The robustness, versatility, and simplicity of the CRISPR–Cas technique have established it as a dominant tool for crop betterment, permitting precise gene regulation, point mutations, gene replacement, knock-in and knock-out strategies, as well as the development of mutant libraries and antiviral breeding approaches. Despite these advances, the widespread translation of CRISPR–Cas technologies from laboratory research to field applications still requires a deeper understanding of the genetic basis of agronomically valuable traits, improved targeting efficiency, and further optimization of delivery systems. Moreover, the integration of GE with functional genomics, proteomics, systems biology, and synthetic biology will accelerate the development of next-generation breeding strategies. Collectively, these advances position CRISPR–Cas GE as a transformative platform for sustainable agriculture, hybrid crop improvement, and global food security.
